# Using a roller pump for establishing extra-corporal membrane oxygenation (ECMO) – technical considerations for times of crisis

**DOI:** 10.1177/0267659121996182

**Published:** 2021-02-26

**Authors:** Mirko Kaluza, Benjamin May, Torsten Doenst

**Affiliations:** Department of Cardiothoracic Surgery, Jena University Hospital, Friedrich-Schiller-University of Jena, Jena, Germany

**Keywords:** veno-venous ECMO, centrifugal pump, roller pump, cardiopulmonary bypass, pulmonary failure

## Abstract

**Objective:**

The COVID-19 pandemic requires thinking about alternatives to establish ECMO when often-limited hardware resources are exhausted. Heart-lung-machines may potentially be used for ECMO but contain roller pumps as compared to centrifugal pumps in ECMO-circuits. We here tested roller pumps as rescue pump for ECMO-establishment.

**Methods:**

We set up in vitro circuits on roller pumps from C5 heart-lung-machine with 5 l/minutes flow. In two series, we placed either PVC or silicon tubing for an ECMO circuit into the roller pump. We assessed the mechanical stress on the tubing (aiming to run the pump for at least 1 week), measured the temperature increase generated by the friction and assessed flow characteristics and its measurement in simulated situations resembling tube kinking and suction.

**Results:**

The roller pumps led to expected and unexpected adverse events. PVC tubing burst between 36 and 78 hours, while silicon tubing lasted for at least 7 days. At 7 days, the silicone tubing showed significant signs of roller pump wear visible on the outside. The inside, however, was free of surface irregularities. Using these tubings in a roller pump led to a remarkable increase in circuit temperature (PVC: +12.0°C, silicone +2.9°C). Kinking or suction on the device caused the expected dramatic flow reduction (as assessed by direct measurement) while the roller pump display continued to show the preset flow. The roller pump is therefore not able to reliably determine the true flow rate.

**Conclusion:**

Roller pumps with silicone tubing but not PVC tubing may be used for running ECMO circuits. Silicone tubing may endure the roller pump shear forces for up to 1 week. Thus, repeated tubing repositioning may be a solution. Circuit heating and substantial limitations in flow detection should increase attention if clinical use in situations of crisis is considered.

## Introduction

The current COVID-19 pandemic places a heavy burden on intensive care resources in severely affected regions, with substantial limitations in the availability of both personnel and equipment.^1,2^ While the majority of ICU patients can be managed with more or less invasive ventilation, there is a smaller fraction of patients where ECMO is required in addition.^3^

The growing experience shows that individual centers or areas may be affected so severely and rapidly, that the often only small number of ECMO units available is quickly exhausted.^4,5^ Yet, the number of available perfusion sets for ECMO is often many fold larger. Thus, a center’s therapeutic potential may not fully be exploited mainly due to the limitation in available ECMO pumps. However, most centers that are heavily affected by the pandemic also experience a substantial decline in standard cardiac surgery procedures, freeing up perfusion resources by availability of the heart-lung-machines. In addition, heart-lung-machines (HLM) contain several pumps per unit and ECMO units only need one pump. Unfortunately, heart lung machines primarily use roller pumps, while for ECMO centrifugal pumps are used. Using roller pumps for longer time frames may result in tubing failure.^6^

We here tested the possibility to use a roller pump as rescue pump for the establishment of ECMO circuits. We specifically wanted to test the durability of standard ECMO perfusion sets in a roller pump by using either classic PVC or silicone tubing and assessed heat development and hemodynamic characteristics.

## Material and methods

An in vitro perfusion circuit was established on a C5 heart-lung-machine (LivaNova) using the large roller pump that normally generates cardiopulmonary bypass flow.

**Figure 1.**
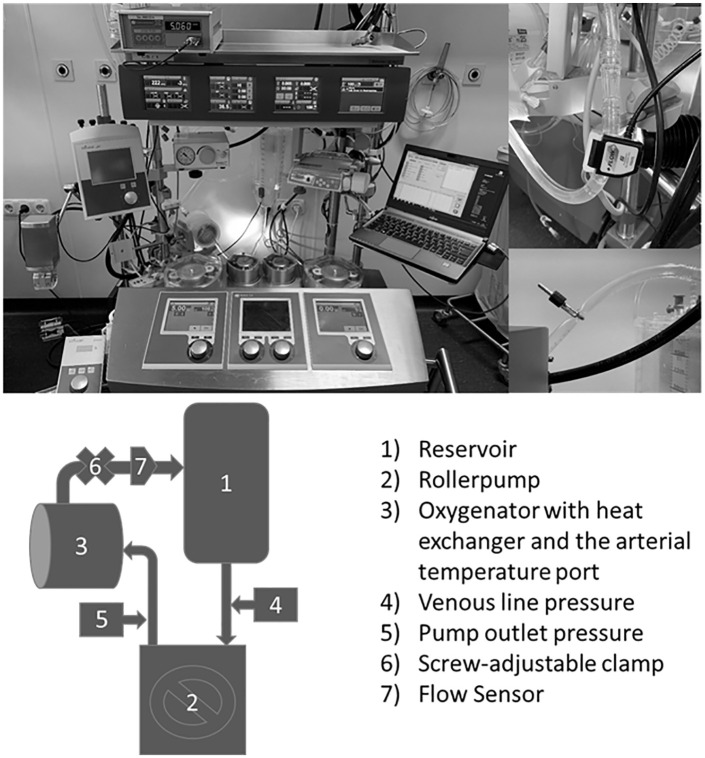
Photograph and schematic illustration of the simulated ECMO circuit.

Figure 1 shows the set-up of the in vitro circuit with the tubing, the roller pump and the oxygenator. The hard-shell reservoir is used to simulate the patient (it is therefore not part of the ECMO circuit). The flow was set to 5 l/minutes. The flow was independently measured with a flow probe connected to the ECMO circuit and displayed with a separate device. The system pressure at the pump outlet was set to 220 mmHg using a screw-adjustable clamp. This pressure corresponds to the mean value in a regular ECMO circuit. The circuits were primed with 850 ml of Ringer’s solution. The following tubing material was used:

Set-up 1: pump tube 1/2″×3/32″ RAUMEDIC ECC noDOP^®^ PVC blood supply tubing.
Set-up 2: 1/2″×3/32″ RAUMEDIC ECC-SIK, silicon tubing.

The roller pump occlusion was set to fully occlusive. The level sensor was installed at the hard-shell reservoir and the level control was connected to the roller pump. Running times of the pumps were recorded using the Data Management System of the heart-lung-machine (DMS, Stöckert Instrumente GmbH Munich). In case of pump tubing rupture, the volume loss decreased the level in the reservoir, the level sensor stopped the pump and the online DMS system recorded the event. From these data we concluded the running time of the pump tubes. Temperature measurement was established at the arterial temperature port of the oxygenator. The oxygenator (Terumo Capiox RX25) also served as system resistance and the heat exchanger of the oxygenator was used to heat the system to the working temperature.

Once operational, we first assessed heat generation through friction of the tubing. The pump was started at room temperature and run for 3.5 hours. Temperature increase was measured. After that, an ECMO heating device (HU 35) was connected to establish perfusate temperature of 36.5°C. Then, durability of tubing roller pump was assessed. We performed three experiments per group and intended to reach 7 days of perfusion. In two cases with silicone tubing perfusion was run for up to 10 days. While running the experimental set-ups for durability, we performed hydrodynamic testing, such as kinking of the tubes or simulated different situations of poor venous drainage. Tubing occlusion was performed either by manual kinking the tubing for “full occlusion” or by using a screw adjustable clamp and compressing the diameter by 50% for “half occlusion.”

## Results

Table 1 shows the running times of the two set-ups and the heat generation within the first 3.5 hours of operation. PVC tubing was unable to withstand the mechanical forces of the roller pump for the intended 7 days of perfusion. The tubing tore along the rolls of the pump after a minimum of 36 hours and a maximum of 78 hours. In addition, the heat generated within the first 3.5 hours was highest with 12.0°C in the PVC group, indicating indirectly the significant mechanical shear forces. The silicone tubing lasted for full 7 days in all three experiments. Heat generation was much lower (between 2.9°C and 3.9°C). The first silicone experiment was terminated after 7 days. The third was terminated after 10 days. However, the second experiment resulted in tube failure after 204 hours (on the ninth day).

**Table 1.** Running times and maximum heat generation in simulated ECMO circuits using a roller pump.

	Tube no.	Running time (in hours)	Maximum heat generation (Δ°C)
Set-up 1 (PVC)	1.1	36	+9.3
	1.2	76	+11.1
	1.3	78	+12.0
Set-up 2 (Silicon)	2.1	168*	+2.9
	2.2	203.6	+3.9
	2.3	240*	+3.7

* Experiment terminated without tubing failure.

**Figure 2.**
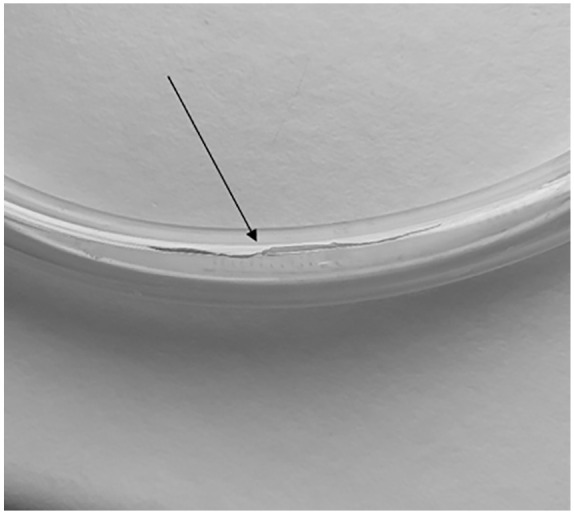
Ruptured PVC tube after 36 hours running time.

**Figure 3.**
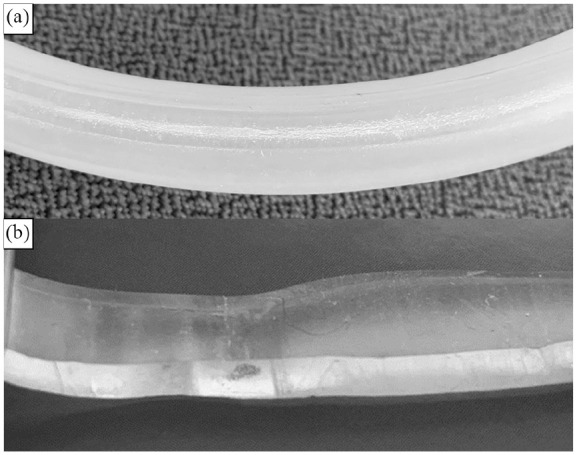
Photographs of a silicon tube after 7 days running time: The outside shows signs of mechanical stress (a) and while the cut open inside maintains a clear surface (b).

Figure 2 shows an example of a PVC tube that tore from the mechanical shear force inside the roller pump. Figure 3 shows signs of mechanical shear forces visible on the outside of the silicone tubing exposed to the pump for 7 days. However, as shown in Figure 3(b), the inside of the tubing was free of macroscopically visible stress, indicating no rubbing off from the inside of the silicone tubes. There was no technical issue with the roller pump and the whole heart-lung-machine performing the continuous work for more than 7 days in any of the experiments.

Table 2 shows the relationship of flow displayed on the roller pump and flow measured independently of the pump under various different conditions simulating possible interfering forces and scenarios in daily practice. Kinking of the arterial line resulted in an increase in arterial line pressure without affecting true flow. However, a complete blockade through full kinking of the tube resulted in pump stop triggered by the pressure sensor of the roller pump. In contrast, kinking of the venous line resulted in stepwise decreases in measured flow while the pump kept on spinning with the display maintaining the 5 l set flow. The same was true for conditions where suction was implemented on the venous reservoir.

**Table 2.** Hemodynamic characteristics of a simulated ECMO circuit with or without tube kinking and suction.

	Pump outlet pressure (mmHg)	Venous line pressure (mmHg)	Arterial line flow^$^ (l/min)	Roller pump display (l/min)
Arterial kinking				
Half^#^	253	−5	5.03	5.0
Full^#^	350^a^	10	0	–
Venous kinking				
Half^#^	103	⩽−250^b^	1.33	5.0
Full^#^	0	⩽−250^b^	0	5.0
Suction^+^				
−50 mmHg	220	−50	5.02	5.0
−85 mmHg	183	−85	4.48	5.0
−120 mmHg	161	−120	3.53	5.0
−200 mmHg	108	−200	1.35	5.0

a Preset point for arterial line pressure to stop pump.

b Maximum displayed negative pressure (−250 mmHg).

$ Measured with a calibrated external outflow measurement device at the arterial line.

# Kinking with hands to simulate kinking situations (which may occur for instance during transportation or with body repositioning of patients in the ICU).

+ Simulation of a low volume status of the patient or kinking of the tubes (negative venous line pressures were predefined).

**Figure 4.**
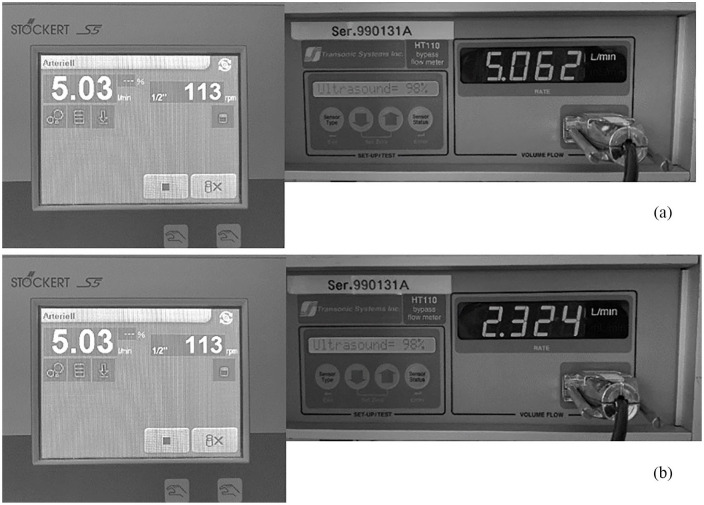
Photographs of the roller pump flow display (left) compared to the individually measured true flow rates (right) under normal conditions (a) and after simulated kinking of the venous tubing or volume shortage (b). Note that the roller pump flow display does not change despite changes in true flow with kinking.

Figure 4 shows a comparison of the set flow at the roller pump as well as the measured flow under normal conditions (a) and when flow was mechanically hindered (b). The reduction in measured flow was not accompanied with changes in the roller pump flow display.

## Discussion

We demonstrate in this in vitro analysis that Roller pumps with silicone tubing but not PVC tubing may be used for running ECMO circuits. Silicone tubing may endure the roller pump shear forces for up to 1 week. Repeated tubing repositioning may be a solution. Circuit heating and substantial limitations in flow detection should be taken into account if clinical use in situations of crisis is considered.

Under normal conditions, centrifugal blood pumps are used to run ECMO circuits. The reason is based on the physical principles that apply to their action. Centrifugal force is used to set the blood into rotation with help of impellers. The rotational speed transfers the energy of the pump to the blood. The generated flow depends on centrifugal head size and is dependent on in- and outflow pressure. Flow is generated with the energy that exceeds the amount needed to generate the tubing pressures. Thus, if pressure increases, flow decreases and if the pump stops, blood is free to flow back according to the prevailing pressure gradients.

In contrast, a roller pump is a robust and efficient pump, where pump flow depends on tube size and rotation speed. There is no backflow while the pump is stopping, for instance in an emergency situation. There is no control or compensation for inflow occlusion or negative pressures. Obstructions in the outflow tubing result in significant pressure increases without limitations in flow over a wide range, because the pump can work against the resistance until its pressure limits will cause it to stop. These differences in design, and especially the lack of mechanical shear forces on the equipment, have made the centrifugal pump the worldwide standard for ECMO circuits.

Our work here suggests that a roller pump may under emergency conditions be used in vivo. This suggestion is supported by the fact that roller pumps are routinely used in pediatric ECMO circuits, although the hemodynamic conditions in children require much less flow.^7–9^ The technical issues such as suction and kinking of tubing are important, have to be recognized by the team but should not limit its use. However, increased attention appears required to run such a system. Including negative pressure measurements for limiting pump speed in case of suction may also be helpful. The connection of the patient to a much larger device (heart-lung-machine) is also likely to limit maneuverability of the patient. In addition, the mechanical shear forces on the tubing appears to be a limiting factor. While PVC tubing may not be used at all because it may not even last for 2 days, silicone tubing appears to be a possible alternative, at least for up to a week. Our findings are also consistent with Peek et al. who assessed durability of three different types of tubing in a roller pump setting and demonstrated tubing rupture between on averaged 7 and 244 hours.^6^ Our conclusions are also supported by our finding that use of PVC resulted in a significantly higher degree of circuit heating under our experimental conditions, which we attribute to the greater degree of friction with the PVC tubing. The silicone tubing did not break and did not show any significant signs of erosions on the inside of the tubing for at least 1 week. However, the outside showed signs of mechanical stress that may raise concern for longer use. This concern is supported by the one breakage on the ninth day of roller pumping. Thus, in case such a set-up is planned to be used in the real practice setting, it may be wise to move the tubing piece subjected to the roller pump every week in order to distribute the shear forces to different parts of the circuit. This suggestion is also consistent with current practice in the pediatric setting.^7–9^ In addition, it is known that centrifugal pumps work more gently on the blood than roller pumps over longer operating times and that there may be an increase in hemolysis, microbubble transmission with longer use of roller pumps.^10–14^

The differences in measured and displayed flow in Table 2 would also have to be considered. In regular ECMO circuits, flow is independently measured and not derived from the pump itself. This principle also applies to roller pumps, with the only difference that the roller pump has a display where flow is shown. For the emergency situation it becomes clear from Table 2 that true flow may not be the same as that displayed on the roller pump. Since under such emergency conditions, it is unlikely that equipment for additional flow measurements is available, these factors would require to pay careful attention to the circuit and judge function also based on the clinical presentation of the patient (e.g. assess the quality of oxygenation).

A final consideration is the durability of roller pump function itself. Build for providing cardiopulmonary bypass support, which is usually limited to several hours, roller pumps may run into problems such as overheating or pump failure when run for days or weeks. We did not observe any problems in the operation of our roller pumps and the whole heart and lung machine during the time frame of our experiments. The manufacturer does not suggest using roller pumps under these conditions. Thus, if this set-up is required in practice is appears wise to have a second roller pump present as backup.

## Conclusion

We demonstrate in this in vitro analysis that roller pumps with silicone tubing but not PVC tubing may be used for running ECMO circuits. Silicone tubing may endure the roller pump shear forces for up to 1 week. Repeated tubing repositioning may be a solution. Circuit heating and substantial limitations in flow detection should be taken into account if clinical use in situations of crisis is considered. Importantly, this last resort-type ECMO circuit appears to require more attention and limits maneuverability of the patient.
